# Yellow Teeth Due to Jaundice

**Published:** 1913-02

**Authors:** 


Yellow Teeth Due to Jaundice.—Thursfield, Proceedings of the Royal Academy of Medicine, reports a case of congenital jaundice, with normal liver and spleen, but black stools, suggesting haematogenic jaundice. The jaundice remained deep for about 10 weeks and then slowly disappeared. At the age of nine months it was noticed that the two lower central incisors were yellow, afterward becoming green, varying in tint from bright to dull.

